# *MIR494* reduces renal cancer cell survival coinciding with increased lipid droplets and mitochondrial changes

**DOI:** 10.1186/s12885-016-2053-3

**Published:** 2016-01-21

**Authors:** Punashi Dutta, Edward Haller, Arielle Sharp, Meera Nanjundan

**Affiliations:** Department of Cell Biology, Microbiology, and Molecular Biology, University of South Florida, Tampa, FL 33620 USA; Department of Integrative Biology, University of South Florida, Tampa, FL 33620 USA

**Keywords:** *MIR494*, Apoptosis, Lipid droplets, LC3B, ATG7

## Abstract

**Background:**

miRNAs can regulate cellular survival in various cancer cell types. Recent evidence implicates the formation of lipid droplets as a hallmark event during apoptotic cell death response. It is presently unknown whether *MIR494*, located at 14q32 which is deleted in renal cancers, reduces cell survival in renal cancer cells and if this process is accompanied by changes in the number of lipid droplets.

**Methods:**

769-P renal carcinoma cells were utilized for this study. Control or *MIR494* mimic was expressed in these cells following which cell viability (via crystal violet) and apoptotic cell numbers (via Annexin V/PI staining) were assessed. By western blotting, *MIR494* cellular responses were validated using *MIR494* antagomir and *Argonaute 2* siRNA. Transmission electron microscopy (TEM) was performed in *MIR494*-transfected 769-P cells to identify ultrastructural changes. LipidTOX green neutral lipid staining and cholesterol measurements were conducted to assess accumulation of lipids droplets and total cholesterol levels, respectively, in *MIR494* expressing 769-P cells. Indirect immunofluorescence and western analyses were also performed to examine changes in mitochondria organization. Co-transfection of *MIR494* mimic with siRNA targeting *LC3B* and *ATG7* was conducted to assess their contribution to formation of lipid droplets in *MIR494*-expressing cells.

**Results:**

*MIR494* expression reduces viability of 769-P renal cancer cells; this was accompanied by increased cleaved PARP (an apoptotic marker) and LC3B protein. Further, expression of *MIR494* increased LC3B mRNA levels and LC3B promoter activity (2.01-fold; 50 % increase). Interestingly, expression of *MIR494* markedly increased multilamellar bodies and lipid droplets (by TEM and validated by LipidTOX immunostaining) while reducing total cholesterol levels. Via immunocytochemistry, we observed increased LC3B-associated endogenous punctae upon *MIR494* expression. In contrast to ATG7 siRNA, knockdown of *LC3B* reduced the numbers of lipid droplets in *MIR494*-expressing cells. Our results also identified that *MIR494* expression altered the organization of mitochondria which was accompanied by co-localization with LC3B punctae, decreased PINK1 protein, and altered Drp1 intracellular distribution.

**Conclusion:**

Collectively, our findings indicate that *MIR494* reduces cell survival in 769-P renal cancer cells which is accompanied by increased lipid droplet formation (which occurs in a LC3B-dependent manner) and mitochondrial changes.

## Background

Activation of cell death pathways including apoptosis, autophagy, and necrosis can oppose cell survival [1]. Since such signaling pathways can be regulated in a miRNA-dependent manner, miRNA expression patterns may provide insight into response to chemotherapeutic agents [2, 3]. Interestingly, apoptosis has recently been shown to be associated with the formation of lipid droplets (LDs) [4, 5]. These subcellular organelles are comprised of neutral lipids (i.e., triacylglycerol and cholesterol esters) that are membrane bound by phospholipids [6]. There are a number of miRNAs with emerging roles in regulating lipid metabolism by targeting genes in lipid pathways [7–13]. Kidney cancer is described as a metabolic disease in which the renal clear cell subtype is characterized by increased lipid droplets [14]; it has yet to be investigated whether miRNAs contribute to metabolic dysregulation in this disease. Interestingly, in this disease, the 14q32 locus is deleted and contains one of the largest miRNA clusters (54 miRNAs) in the human genome and is located within the DLK1-DIO3 region [15]. Amongst the miRNAs located at this region, *MIR494*, located at ch14:101029634 – ch14:101029714, has thus far been already implicated in altering epithelial-mesenchymal transition (EMT) [16], senescence [17, 18], cell cycle arrest [19], and apoptosis [20] in a few cancer cell types. Whether *MIR494* alters renal cancer cell survival and lipid droplet formation is presently unknown.

Herein, we demonstrate that expression of *MIR494* in the 769-P renal cancer cell line reduces cellular viability coinciding with increased LC3B RNA and protein. We noted increased lipid droplets in *MIR494* expressing cells (via TEM and cellular staining with LipidTOX) which was dependent on LC3B protein expression. In addition, *MIR494* expression led to mitochondrial changes that involved changes in Drp1 localization and reduced PINK1 protein, molecules involved in altering mitochondrial structural patterns. Collectively, these findings implicate *MIR494* expression in reducing renal cancer cell survival accompanied by increased lipid droplet formation and mitochondrial changes.

## Methods

### Ethics approval

No animal or human specimens were used in this study. The cell lines used (as described below) are de-identified and cannot be linked back to human subjects. The studies reported in this manuscript were submitted to the IRB at the University of South Florida. They provided official assessment of Not Human Subjects Research Determination (IRB#: Pro00024882). The IRB Chairperson is Dr. E Verena Jorgensen at the University of South Florida Institutional Review Board.

### Cell lines

769-P renal carcinoma cells were obtained from ATCC (Manassas, VA). Normal immortalized (LTAg/hTERT) ovarian surface epithelial cells (T80) were kindly provided by Dr. Gordon Mills (MD Anderson Cancer Center, Houston, Texas). 769-P and T80 cells were cultured in RPMI 1640 (Hyclone, Fisher Scientific, Pittsburgh, PA) supplemented with 8 % FBS and penicillin/streptomycin. Cells were maintained in a 37 °C humidified incubator containing 95 % air and 5 % CO_2_. All cell lines used in this study were authenticated by STR profiling (Genetica DNA Laboratories Inc., Cincinnati, OH) and mycoplasma tested as negative.

### Preparation of As_2_O_3_ and cisplatin for T80 cell treatment

As previously described, As_2_O_3_ was dissolved in NaOH followed by dilution with Nanopure water [21]. A stock solution of 5 mM was prepared and used at a final concentration of 2, 5, 10, 25, or 50 μM (Sigma-Aldrich, St. Louis, MO). Cisplatin (Calbiochem, #232120) was dissolved in phosphate-buffered saline (PBS) at a stock concentration of 6.7 mM and used at a final concentration of 100 μM. T80 cells were seeded at 250,000 cells/well in 6-well plates. Following overnight adherence, they were treated with the above mentioned doses of As_2_O_3_ for 18 h and cisplatin for 12, 18, and 24 h.

### miRNA and siRNA transfections

Cells were seeded at 250,000 cells/well in 6-well plates. Following overnight adherence, they were transfected with control *MIR* (mirVana miRNA mimic Negative control 1, #4464058, Life Technologies, Grand Island, NY) or *MIR494* (mirVana miRNA mimic, hsa-miR-494-3p, #4464066 (ID MC12409), Life Technologies, Grand Island, NY) (final concentration of 200 pmol) using Fugene HD (Promega, Madison, MI). Cells were recovered 24 h post-transfection. Protein lysates were harvested 96 h post-transfection.

For transfection of siRNA (*Ago2*, L-004639-00; *ATG7*, L-020112-00; *LC3B*, L-012846-00; non-targeting ON-TARGETplus control (D-001810-10-20), Dharmacon, Lafayette, CO), in combination with miRNA [22], 769-P cells were seeded at 750,000 cells/well. Following 24 h, an initial round of siRNA treatment was performed using a dose of 50 nM. Another round of siRNA transfection (50 nM) was performed on the following day. Twenty-four hours later, cells were recovered and then re-seeded at 250,000 cells/well. On the successive day, cells were transfected with control *MIR* or *MIR494* (200 pmol). Cell lysates were harvested 72 h post-*MIR* transfection for western analyses, immunofluorescence staining, or annexin V-FITC/PI staining. For LipidTOX neutral lipid staining, cells were re-seeded on glass coverslips following two rounds of siRNA transfection, as described above.

### Protein harvest and western blotting

Cells were incubated in lysis buffer (1 % Triton X-100, 50 mM HEPES, 150 mM NaCl, 1 mM MgCl_2,_ 1 mM EGTA, 10 % glycerol, and protease inhibitor cocktail) for 1 h at 4 °C. Cell lysates were harvested by scraping and centrifuged at 14,000 rpm for 10 min at 4 °C. Normalized samples (using the BCA assay (Fisher Scientific, Pittsburgh, PA)) were run on SDS-PAGE gels and transferred to polyvinylidene fluoride (PVDF) membranes for western blotting. Bound antibody was detected using enhanced chemiluminescence reagent followed by exposure to film. Primary antibodies were used at the following dilutions and obtained from the following sources: Ago2 rabbit monoclonal (#2897, 1:500), caspase 2 mouse monoclonal (1:1000), caspase 3 rabbit monoclonal (1:1000), caspase 8 mouse monoclonal (1:1000), and caspase 9 mouse monoclonal (1:1000) (Initiator caspases sampler kit #12675), Drp1 rabbit monoclonal (#8570, 1:1000), GAPDH rabbit monoclonal (#2118, 1:5000), LC3B rabbit polyclonal (#2775, 1:1000), pan-actin rabbit polyclonal (#4968, 1:1000), PARP rabbit polyclonal (#9542, 1:1000), and PINK1 rabbit monoclonal (#6946, 1:500) antibodies were obtained from Cell Signaling Technology (Danvers, MA). ATG7 rabbit polyclonal antibody (PM039, 1:1000) was obtained from MBL International Corporation (Woburn, MA).

### RNA isolation and quantitative PCR

769-P cells were seeded at 250,000 cells/well and transfected with miRNA following overnight adherence. Twenty-four hours post-transfection, cells were trypsinized and re-seeded at 250,000 cells/well and then at 96 h post-transfection, total RNA isolation was then carried out using the RNeasy Mini Kit from Qiagen (Valencia, CA). Real-time PCR was performed using the One-Step PCR Taqman Master Mix (Applied Biosystems, Grand Island, NY). Probes/primers for *LC3B* were obtained from Applied Biosystems (Assays-on-Demand (Hs00797944_s1)). β-actin was used as the endogenous control. PCR cycle conditions and analyses were performed as reported previously [21].

### miRNA isolation and quantification

The mirVana miRNA isolation kit from Ambion (Grand Island, NY) was utilized for total RNA isolation (according to the manufacturer’s protocol). The RNA concentrations were assessed using NANOdrop. The TaqMan miRNA probe-based qRT–PCR reaction (Taqman MicroRNA Assays, Applied Biosystems, Grand Island, NY) was performed in reaction buffer containing dNTPs and reverse transcriptase enzyme (7 μl). The total reaction volume was 15 μl (5 μl RNA and 3 μl probes/primers). The reaction conditions for RT were as follows: 30 min, 16 °C; 30 min, 42 °C; 5 min, 85 °C. The PCR reaction conditions were as follows: 10 min, 95 °C; 50 cycles (Denature: 15 s, 95 °C; Anneal: 60 s, 60 °C). The RNA concentration utilized was 500 μg/μl in 20 μl total reaction volume (TaqMan MicroRNA Assay, RT product, TaqMan Universal PCR Master Mix). The relative miRNA levels were calculated using the comparative C_T_ method. The probes/primers utilized for the reverse transcription and PCR reactions for *MIR494* were RT:002365, TM:002365 and for RNU6B were RT:001093, TM:001093.

### Cell viability assay

769-P cells were seeded at 250,000 cells/well in 6 well plates. Transfection with *MIR494* or control *MIR* was performed as described above. Twenty-four hours post-transfection, cells were re-seeded into 96 well plates at 2500 or 5000 cells/well. At 120 h post-transfection, media was removed and cells stained with crystal violet for 15 min at room temperature. The cells were washed with nanopure water and after overnight drying, Sorenson’s buffer was added, shaken for 2 h at room temperature, and then read at 570 nm using a Biotek plate reader.

### Apoptosis assay

For assessment of apoptosis, annexin V-PI staining was performed following manufacturer’s instructions (#PF032, Calbiochem, San Diego, CA). Briefly, cells were seeded at 250,000 cells/well in 6-well plates. Following *MIR494* or control *MIR* transfections, both floating and adherent (by trypsinization) cell populations were collected and pelleted 96 h post-miRNA transfection. Cell pellets were then resuspended in PBS followed by the addition of annexin V and PI, after which the samples were analyzed by flow cytometry (Karoly Szekeres, College of Medicine, Flow Cytometry Core, University of South Florida, Tampa, Florida).

For *ATG7 or LC3B* siRNA, 769-P cells were seeded at 750,000 cells/well. Following overnight adherence, two successive rounds of siRNA knockdown was performed (50 nM). Twenty-four hours later, cells were recovered and then re-seeded at 250,000 cells/well. On the successive day, cells were transfected with control *MIR* or *MIR494* (200 pmol). Seventy-two hours post-mimic transfection, cells were processed for annexin V-PI staining as described above.

### Indirect immunofluorescence

769-P cells were seeded at 250,000 cells/well. Following overnight attachment, cells were transfected with control *MIR* or *MIR494* as described above. Twenty-four hours post-transfection, cells were trypsinized and re-seeded on glass coverslips at 150,000 cells/well. Ninety-six hours post-transfection, cells were fixed using 4 % formaldehyde for 30 min at room temperature (this method of fixation was used for AIF rabbit monoclonal antibody (Cell Signaling Technology, #5318, 1:400), cytochrome c mouse monoclonal antibody (Cell Signaling Technology, #12963, 1:250), and Drp1 rabbit monoclonal antibody (Cell Signaling Technology, #8570, 1:50)), or fixation in 100 % cold methanol for 15 min at −20 °C (for LC3B rabbit polyclonal antibody, Cell Signaling Technology, #2775, 1:400 dilution). For experiments involving co-staining of LC3B and cytochrome c, cells were first fixed with 4 % formaldehyde for 15 min at room temperature followed by fixation in 100 % cold methanol for 15 min at −20 °C.

769-P cells were seeded onto glass coverslips at 1 million cells/well in 6-well plates. Cell were then fixed and stained the following day with AIF rabbit monoclonal antibody (Cell Signaling Technology, #5318, 1:400) or COXIV monoclonal antibody (Cell Signaling Technology, #4850, 1:250).

T80 cells were seeded at 500,000 cells/well in 6 well plates onto glass coverslips. Twenty-four hours post-seeding, treatment with cisplatin was initiated. Cells were then fixed and stained with AIF rabbit monoclonal antibody as described above.

The mitochondrial structural patterns were divided into four categories: (1) tubular elongated, (2) tubular shortened, (3) tubular shortened fragmented, and (4) fragmented mitochondria. Cells were counted, assigned to these four categories, LC3B punctae status recorded, and quantified accordingly.

Co-localization of cytochrome c with Drp1 as well as LC3B with cytochrome c were performed using Volocity 3D Imaging Software (version 6.3) from PerkinElmer (Waltham, MA). Thresholds were set for individual channels and Pearson coefficients averaged for each set of replicates. Data analyzed for Fig. 5b and f are shown in Fig. 5c and g as Pearson coefficients which are expressed as averages ± standard deviation.

### mCherry-GFP-LC3B autophagic flux assay and image J macro analysis

The 769-P cells stably expressing mCherry-GFP-LC3B (retroviral pool 1 and 2) were seeded at 250,000 cells/well on glass coverslips. Cells were transfected with control *MIR* and *MIR494*, following overnight adherence and at ninety-six hours post-transfection, cells were fixed, blocked, and coverslips mounted on glass slides with DAPI mounting media.

Analysis of autophagic flux was performed using Image J (http://imagejdocu.tudor.lu/doku.php?id=plugin:analysis:colocalization_analysis_macro_for_red_and_green_puncta:start). Briefly, a total of 10 pictures were captured for each sample (120 pictures) using a Perkin Elmer Confocal Spinning Disc Microscope (CMMB Core Facility, University of South Florida, Tampa, Florida) followed by Image J Macro analysis for each of the images captured. This program was used to quantify the green, red, and merged (yellow) punctae.

### Transmission Electron Microscopy (TEM) for ultrastructural analysis

Duplicate 100 mm dishes of 769-P cells expressing control or *MIR494* were submitted for transmission electron microscopy. The cells were fixed *in situ* with 2.5 % phosphate buffered glutaraldehyde, post-fixed with 1 % osmium tetroxide, scraped from the dishes, the duplicate dishes were pooled, and the cells were pelleted by centrifugation and embedded in 3 % agarose. Blocks were produced from the agarose of control and treated cells, which were dehydrated in a graded series of acetone dilutions, cleared in propylene oxide and embedded in LX 112 epoxy resin (Ladd Research Industries, Williston, VT). Following polymerization, ultrathin sections of the samples were obtained, stained with 8 % uranyl acetate and Reynold’s lead citrate, examined and photographed on an FEI Morgagni TEM (FEI, Hillsboro, OR) at 60 kV.

### LipidTOX neutral lipid staining

769-P cells were seeded at 250,000 cells/well. Following overnight attachment, cells were transfected with control *MIR* or *MIR494* as described above. Twenty-four hours post-transfection, cells were trypsinized and re-seeded on glass coverslips at 150,000 cells/well. When experiments required co-transfection of siRNA and miRNA, 250,000 cells were re-seeded after the co-transfection was completed and processed at 72 h post-mimic transfection. Ninety-six hours post-transfection, cells were fixed using 4 % formaldehyde for 30 min at room temperature, followed by a PBS wash and LipidTOX green neutral lipid staining (#H34475, Life Technologies) at a 1:200 dilution in PBS for 1 h. Coverslips were mounted on glass slides along with DAPI mounting media. Imaging was carried out using a Perkin Elmer Confocal Spinning Disc Microscope (CMMB Core Facility, University of South Florida, Tampa, Florida).

### Cholesterol measurements

Cell protein lysates were collected and normalized as described above. The Amplex red cholesterol assay kit (#A12216, Life Technologies) was used to measure total cholesterol content. The samples were diluted in 1X reaction buffer provided with the kit at a 2:3 ratio. Fluorescence measurements were captured on a Biotek plate reader.

### LC3B 3′-UTR and promoter luciferase assays

T80 cells were seeded at 250,000 cells/well. Following overnight adherence, cells were transfected using Fugene HD with 1 μg of pEZX-MT01 plasmid harboring 3′-UTR of *LC3B* downstream of firefly luciferase (LC3B, HmiT019948-MT01) and 200 pmol of control or *MIR494*. Twenty-four hours post-transfection, cells were washed in PBS and then the assay was performed following the manufacturer’s instructions (#LPFR-M010, GeneCopoeia, Rockville, MD).

T80 cells were seeded at 250,000 cells in 6-well plates. Following overnight attachment, cells were transfected using Fugene HD with 1 μg of pLightSwitch promoter plasmid (Switchgear Genomics, Carlsbad, CA) harboring the LC3B promoter upstream of RenSP (#32031) with 200 pmol of control or *MIR494*. Twenty-four hours post-transfection, cells were washed in PBS and then the assay was conducted following the manufacturer’s instructions for the pLightSwitch Luciferase Assay system.

### Statistical analyses

The number of independent replicates are as specified in the Figure Legends. Error bars represent standard deviations and p-values (generated using Graphpad Prism software) were derived by performing the standard student’s t-test (**** = *p* ≤ 0.0001, *** = *p* ≤ 0.001, ** = *p* ≤ 0.01, * = *p* ≤ 0.05 and ns = not significant (*p* > 0.05)).

## Results

### *MIR494* modulates cell viability by altering the apoptotic response and LC3B levels

**Fig. 1:**
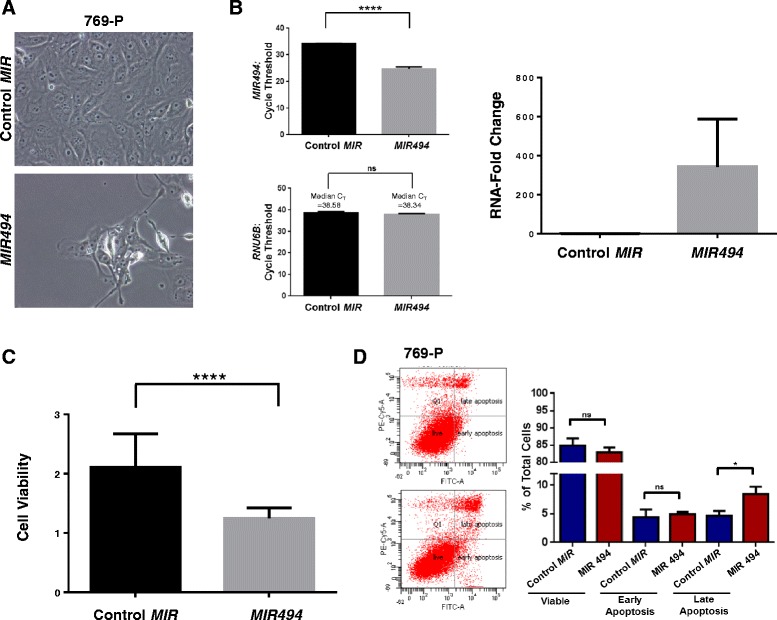
*MIR494* modulates cell viability by altering the apoptotic response and LC3B levels. **a** Light microscope images of 769-P cells expressing *MIR494* or control *MIR* were captured 96 h post-transfection. Representative images at 40× magnification are presented. **b** miRNA isolation and quantification of *MIR494* was performed in 769-P cells expressing control or *MIR49*4. Cycle threshold changes (left panel) and RNA-fold changes (right panel) are presented. Three independent experiments were performed. **c** 769-P cells expressing *MIR494* or control *MIR* were re-seeded into 96-well plates; following 96 h post-transfection, cell viability was assessed. A total of five independent replicates were performed. **d** Annexin V-PI staining was performed in 769-P cells expressing *MIR494* or control *MIR* at 96 h post-transfection. Raw data plots are shown as log fluorescence values of annexin V-FITC and PI on the X and Y axis, respectively. The percentage of viable, early apoptotic, and late apoptotic cells are shown. Three independent replicates were performed

To assess the functional changes elicited by *MIR494* expression in 769-P cells, we initially examined changes in cellular morphology via light microscopy ninety-six hours post *MIR494* transfection. As shown in Fig. 1a, we observed a reduction in cell density and large cytoplasmic vacuoles in 769-P cells expressing *MIR494*. We assessed cellular viability (Fig. 1c) and quantified the miRNA level of *MIR494* following expression (Fig. 1b). As shown in Fig. 1d, *MIR494* expression induced an increase in late apoptotic cells in the 769-P cell line.

**Fig. 2:**
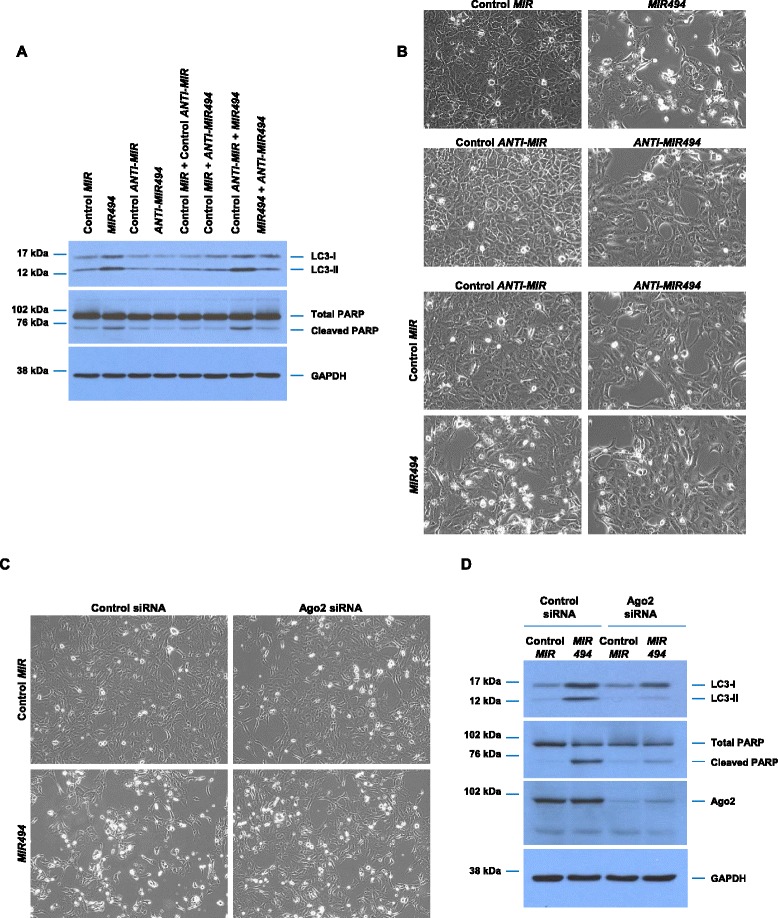
Validation of *MIR494* cellular responses. **a** 769-P cells were seeded at 50,000 cells/well in a 24-well plate. Twenty-four hours post-seeding, cells were transfected with control mimic, *MIR494* mimic, control antagomir, *MIR494* antagomir in combination as indicated. Ninety-six hours post-transfection, protein lysates were collected, samples run on a SDS-PAGE gel, and analyzed via western blotting using the indicated antibodies. Three independent experiments were performed. **b** Representative light micrograph images are shown. **c** Representative light micrographs of 769-P cells treated with *Ago2* siRNA and expressing *MIR494* or control *MIR* are presented. **d** Protein lysates isolated from 769-P cells treated with *Ago2* siRNA and expressing *MIR494* or control *MIR* were analyzed by western blotting using the indicated antibodies. Three independent experiments were performed

These changes in apoptotic response were validated via western analysis by assessing PARP cleavage (an apoptotic marker) which increased in *MIR494* expressing cells. In addition, we assessed LC3B expression, a marker of the autophagic pathway which regulates cell survival responses, which also markedly increased (Fig. 2a). To ensure that the *MIR494*-mediated effect on cleaved PARP and LC3B were specific to the miRNA, we first tested the effect of an antagomir targeting *MIR494* in 769-P cells. Following ninety-six hours of *MIR494* expression in the presence or absence of anti-*MIR494*, we noted that addition of antagomir to *MIR494* expressing cells increased cell density compared to cells only expressing *MIR494* (Fig. 2b). Indeed, cells treated with anti-*MIR494* had a marked reduction in cleaved PARP (Fig. 2a). In addition, we noted that the LC3B levels reduced to baseline levels in the presence of anti-*MIR494* compared to *MIR494* expressing cells (Fig. 2a). In addition, we performed knockdown of *Argonaute 2* (*Ago2*), a protein involved in the formation of the RISC (RNA-induced silencing complex) complex essential for binding to target mRNA, in the absence or presence of *MIR494*. As shown in Fig. 2c and d, with >80 % reduction in Ago2 protein, reduction of Ago2 in cells expressing *MIR494* increased cell density compared to cells with wild type Ago2 expression in the presence of *MIR494* (Fig. 2c). Western blot analyses showed a marked reduction in cleaved PARP and LC3B levels compared to cells expressing *MIR494* with wild type *Ago2* levels.

**Fig. 3:**
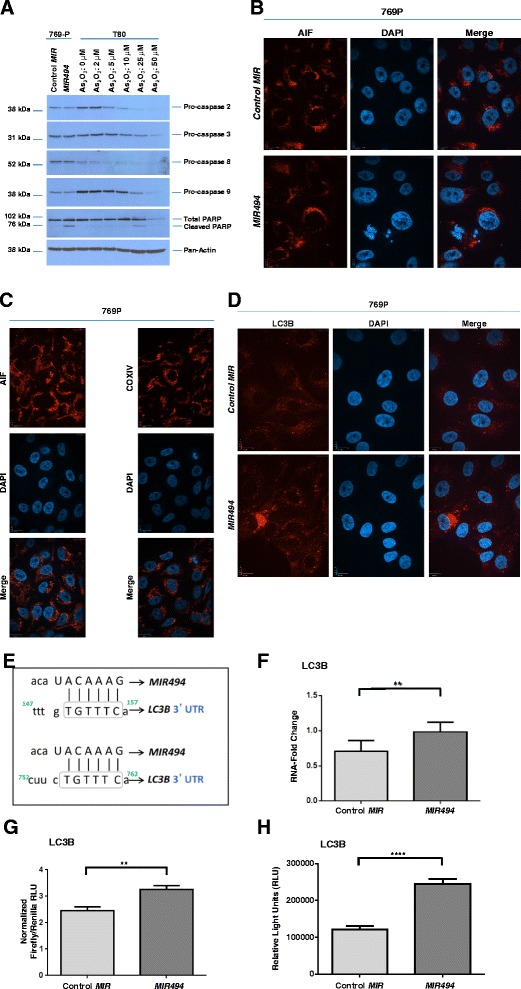
*MIR494* induces LC3B mRNA expression and LC3B-associated punctae. **a** T80 cells were seeded at 250,000 cells/well. Twenty-four hours post-seeding, cells were treated with the indicated doses of As_2_O_3_ for 18 h, after which protein lysates were collected. Samples were run on a SDS-PAGE gel and analyzed via western blotting using the indicated antibodies. Two independent experiments were performed. **b** Indirect immunofluorescence was performed on 769-P cells transfected with *MIR494* or control *MIR* at 96 h post-transfection for AIF. Three independent experiments were performed. Representative images are presented. **c** Indirect immunofluorescence was performed on 769-P cells for AIF or COXIV. Representative images are presented. **d** 769-P cells expressing *MIR494* were subjected to immunofluorescence staining for LC3B. Two independent experiments were performed. Representative images are presented. **e** The schematic depicts *MIR494* binding sites in the 3′-UTR of *LC3B* (2 imperfect binding sites). Grey boxes indicate the binding region on the mRNA transcript of LC3B. **f** Total RNA was isolated from 769-P cells expressing *MIR494* or control *MIR* and used for real-time PCR. Relative RNA-fold changes are presented for *LC3B*. Three independent experiments were performed. **g** T80 cells were transfected with pEZX-MT01 plasmid harboring the 3′-UTR of *LC3B* downstream of the luciferase gene in the absence or presence of *MIR494*. Three independent experiments were performed. **h** T80 cells were transfected with pLightSwitch plasmid harboring the promoter of *LC3B* upstream of the luciferase gene in the absence or presence of *MIR494*. Three independent experiments were performed

To further define the apoptotic pathway induced by *MIR494* in 769-P cells, we assessed caspase- and AIF-dependency. Via western analyses, we examined the activation status of both initiator and executioner caspases in the absence or presence of *MIR494* expression. In contrast to T80 cells (a normal immortalized ovarian cell line) [23] treated with increasing doses of arsenic trioxide (As_2_O_3_) which showed a marked reduction in expression of pro-caspase 2, 3, 8, and 9 with increasing doses of As_2_O_3_ (our previous findings support these results [21]), 769-P cells expressing *MIR494* did not elicit any reproducible changes in expression of the pro-caspases assessed (Fig. 3a). Since AIF is reported to be involved in caspase-independent apoptosis by translocating from the mitochondria to the nucleus to induce DNA fragmentation [24], we performed immunofluorescence staining for AIF in 769-P cells expressing *MIR494*. Based on COXIV immunostaining (a mitochondrial marker) (Fig. 3c) in parental 769-P cells, it would appear that AIF remains associated with the mitochondria under baseline conditions. As shown in Fig. 3b, we did not observe nuclear localization of AIF upon *MIR494* expression at 96 h post-transfection (or in T80 cells treated with cisplatin (results not shown)). Collectively, these findings suggest that *MIR494* mediates an apoptotic response that does not involve activation of caspase 2, 3, 8, or, 9 or localization of AIF to the nuclear compartment.

As shown in Fig. 3d, there was a marked increase in both the size and number of LC3B endogenous punctae with *MIR494* expression in the absence of changes in autophagic flux (results not shown). We noted 2 imperfect *MIR494* binding sites in the 3′-UTR of LC3B (Fig. 3e). To assess whether *MIR494* alters the LC3B RNA transcript level, we performed real-time PCR analysis using RNA isolated from *MIR494* expressing cells and determined that LC3B RNA was increased (1.39-fold; 28 % increase) upon *MIR494* expression relative to control cells (Fig. 3f). Next, to assess whether LC3B could be a target of *MIR494*, we performed a 3′-UTR luciferase assay. As shown in Fig. 3g, expression of *MIR494* increased (1.33-fold; 25 % increase) luciferase activity in cells transfected with a plasmid containing the 3′-UTR of *LC3B*. Since miRNAs have recently been implicated in increasing RNA transcript levels via binding to gene promoter elements, *MIR494* could therefore be mediating its effect via the promoter of LC3B. Therefore, we assessed whether *MIR494* could modulate LC3B promoter activity. As shown in Fig. 3h, we noted that expression of *MIR494* increased LC3B promoter activity 2.01-fold (50 % increase). These results suggest that LC3B may be a downstream target of *MIR494*. Additionally, since LC3B is involved in autophagy, we also investigated whether *MIR494* led to any change in autophagic flux. However, we did not identify autophagic flux changes with *MIR494* expression in 769-P cells (results not shown).

### Increased lipid droplets in *MIR494*-expressing cells

**Fig. 4:**
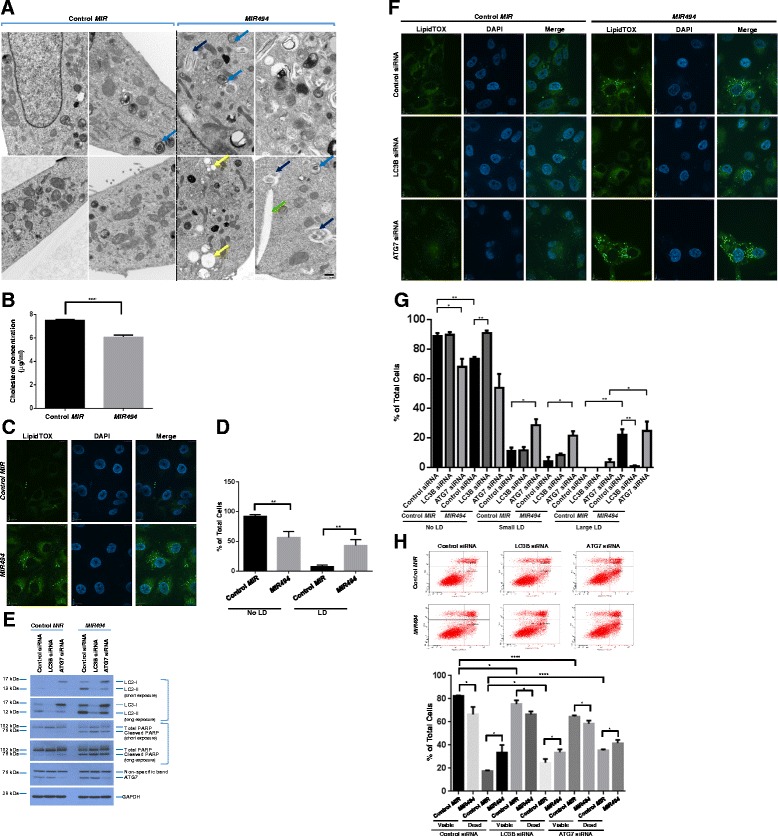
*MIR494* increases lipid droplets in an LC3B-dependent and ATG7-independent manner. **a** TEM images captured from *MIR494* or control *MIR* transfected 769-P cells. Yellow arrowheads indicate lipid droplets, green arrowheads indicate cholesterol clefts, blue arrowheads indicate multilamellar bodies, and dark blue arrowheads indicate lipid whorls (autophagosomes). **b** 769-P cells expressing *MIR494* or control *MIR* were utilized for cholesterol measurements ninety-six hours post-transfection. Three independent experiments were performed. **c** 769-P cells expressing *MIR494* or control *MIR* were re-seeded at 150,000 cells/well on glass coverslips. Ninety-six hours post-transfection, cells were fixed and stained with green neutral lipid stain. Representative images from three independent experiments are presented. **d** Graphical quantification of the data obtained from (**c**). **e** Protein lysates isolated from 769-P cells treated with *LC3B* or *ATG7* siRNA in the absence or presence of *MIR494* or control *MIR* were analyzed by western blotting using the indicated antibodies. Three independent experiments were performed. **f** 769-P cells treated with *LC3B* or *ATG7* siRNA were re-seeded at 250,000 cells/well on glass coverslips. Twenty-four hours post re-seeding, *MIR494* or control *MIR* transfection was performed. Seventy-two hours post-transfection, cells were fixed and stained with green neutral lipid stain. Representative images from three independent experiments are presented. **g** Graphical quantification of the data presented in (**f**) is shown. **h** 769-P cells treated with *LC3B* or *ATG7* siRNA in the presence or absence of *MIR494* were analyzed by annexin V/PI staining. Raw data plots are shown as log fluorescence values of annexin V-FITC and PI on the X and Y axis, respectively. The percentage of viable and dead cells are shown. Three independent experiments were performed

Since it has been reported that the apoptotic response is associated with the formation of lipid droplets [4], we next performed transmission electron microscopy (TEM) to identify ultrastructural changes including the formation of lipid droplets in *MIR494* expressing cells. As shown in Fig. 4a, we noted a marked increase in the numbers of lipid droplets, cholesterol clefts, and multilamellar bodies in *MIR494* expressing cells.

To validate these changes observed by TEM, we measured total cellular cholesterol levels. However, in contrast to the TEM which showed increased cholesterol clefts, *MIR494* expression was found to reduce total cellular cholesterol levels compared to control cells (Fig. 4b). This response is similar to that reported for chemotherapeutic agents that deplete intracellular cholesterol which sensitizes cancer cells to cell death [25]. We then utilized LipidTOX immunofluorescence stain to validate whether *MIR494* alters lipid droplet numbers and/or size. As shown in Fig. 4c and d, we observed a marked increase in lipid droplets upon *MIR494* expression. Since LC3B and ATG7 are associated with the outer surface of lipid droplets [26] and deletion of ATG7 in a mouse model promotes lipid accumulation [27], we next assessed whether these two molecules may contribute to *MIR494-*mediated formation of lipid droplets. Thus, we performed siRNA-mediated knockdown of *LC3B* and *ATG7* in the absence or presence of *MIR494* expression. By western analyses, we noted a marked reduction in LC3B levels with siRNA targeting *LC3B* while *ATG7* knockdown markedly altered the ratio of LC3-I/II (Fig. 4e). LipidTOX neutral lipid staining in cells transfected with control, *LC3B*, or *ATG7* siRNA in the absence or presence of *MIR494* expression is shown in Fig. 4f and g. Knockdown of *ATG7* alone led to an increase in the number of lipid droplets which was further increased upon expression of *MIR494*. Compared to control siRNA in the presence of *MIR494*, *LC3B* siRNA with *MIR494* expression significantly reduced the numbers of lipid droplets. These results indicate that LC3B contributes to *MIR494*-mediated increase in lipid droplet formation while this process is independent of ATG7. With *MIR494* expression, we also noted an increase in cleaved PARP in control, *LC3B*, and *ATG7* siRNA treated cells. However, via annexin V/PI staining, we did not identify any large changes in dead cells upon *MIR494* expression with *LC3B* or *ATG7* knockdown, compared to control siRNA (Fig. 4h). This result suggests that LC3B or ATG7 only contribute a small aspect of the *MIR494* apoptotic response.

### Mitochondrial changes are observed with *MIR494* expression

**Fig. 5:**
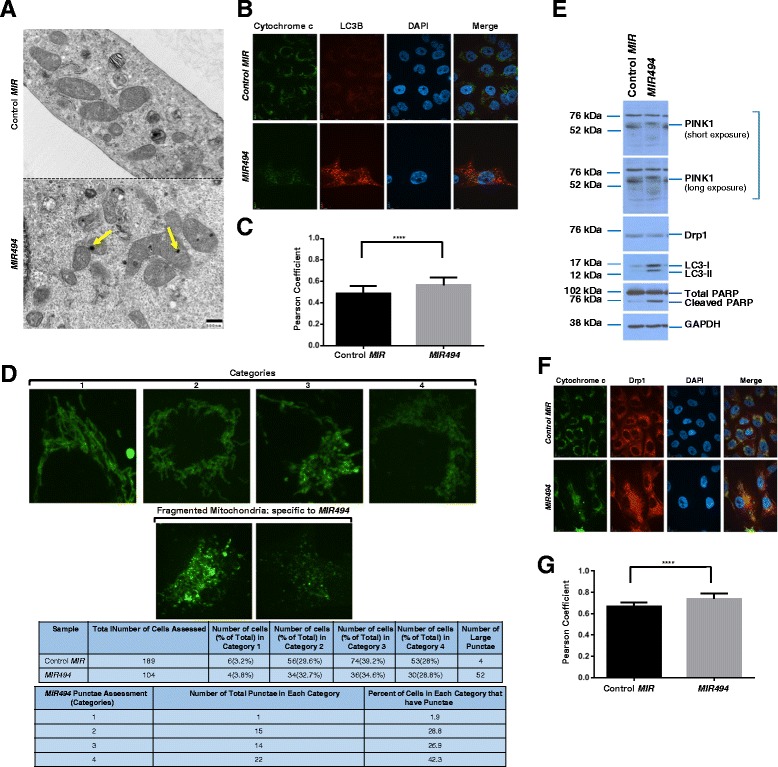
*MIR494* expression leads to mitochondrial changes. **a** TEM images were captured from control or *MIR494* transfected 769-P cells. Yellow arrowheads indicate electron dense regions in the mitochondria. **b** Indirect immunofluorescence was performed on 769-P cells transfected with *MIR494* or control *MIR* at 96 h post–transfection for LC3B and cytochrome c. Three independent experiments were performed. Representative images are presented. **c** Pearson coefficients are shown for data presented in (**b**). **d** Mitochondrial patterns were segregated into four distinct categories as described in Results. The quantified data is presented in tabular form. **e** Protein lysates collected from *MIR494* or control *MIR* expressing 769-P cells were re-run on a SDS-PAGE gel and analyzed via western blotting using the indicated antibodies. Three independent experiments were performed. **f** Indirect immunofluorescence was performed on 769-P cells transfected with *MIR494* or control *MIR* at 96 h post-transfection for Drp1 and cytochrome c. Three independent experiments were performed. Representative images are presented. **g** Pearson coefficients are shown for data presented in (**f**)

Since mitochondria undergo dramatic structural changes during the apoptotic response and are also involved in the uptake of fatty acids from lipid droplets [28], we next assessed mitochondrial changes upon *MIR494* expression in 769-P cells. By TEM analyses, we noted an electron dense region in the mitochondria of *MIR494* expressing cells relative to control cells (Fig. 5a). To investigate the nature of these mitochondrial changes, we performed immunofluorescence staining with cytochrome c. Since mitochondria undergo dynamic morphological changes, we segregated the structural patterns of cytochrome c into four categories: (1) tubular elongated, (2) tubular shortened, (3) tubular shortened fragmented, and (4) fragmented mitochondria. We captured images of control and *MIR494* expressing cells and classified the cytochrome c mitochondrial staining pattern into these four categories. In *MIR494* expressing cells, we observed a marked increase in category 3 and 4 mitochondrial patterns (fragmented mitochondria). Furthermore, by assessing endogenous LC3B co-localization with cytochrome c via immunofluorescence staining, we determined that there was increased LC3B co-localization to category 3 and 4 fragmented mitochondria (Fig. 5b and c (Pearson coefficients)). These results suggest that *MIR494* expression may alter mitochondrial structures which are associated with LC3B punctae.

Proteins involved in mitochondrial dynamics include PTEN-induced putative kinase 1 (PINK1, involved in phosphorylation and recruitment of Parkin to mitochondria during mitophagy) [29] and Dynamin-related protein 1 (Drp1, involved in mitochondrial fission events) [30]. Since we observed that *MIR494* expression increased mitochondrial fragmentation [31], we next assessed changes in protein expression of PINK1 or Drp1 by western analyses. As shown in Fig. 5d, we observed a decrease in PINK1 levels in the absence of changes in Drp1 protein in *MIR494* expressing cells. Since reduction of PINK1 protein leads to mitochondrial fission in a Drp1-dependent manner [29], we then assessed whether Drp1 localization was altered with *MIR494* expression. As shown in Fig. 5e and f (Pearson coefficients), co-localization of Drp1 with cytochrome c was increased in *MIR494* expressing cells. Additionally, since PINK1 loss is reported to increase oxidative stress and thus mitochondrial fission [29], we then assessed superoxide levels using MitoSOX in *MIR494* expressing cells. However, we did not identify any significant or reproducible changes in superoxide levels in *MIR494* expressing cells compared to control cells (results not shown). Collectively, these results suggest that the mitochondrial fragmentation observed with *MIR494* expression may be due to reduction in PINK1 protein and Drp1 recruitment to mitochondria.

## Discussion

**Fig. 6:**
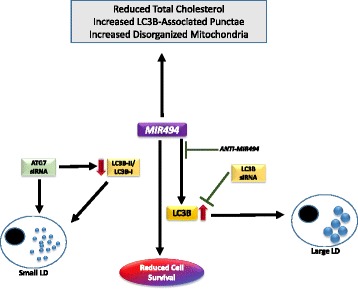
Model of *MIR494* cellular responses in 769-P cells. *MIR494* induces increase in cleaved PARP and LC3B in 769-P cells. This response was Ago2-dependent and was reversed upon addition of anti-*MIR494. MIR494* expression led to reduction in total cholesterol levels, mitochondrial changes, LC3B punctae changes, and a significant increase in lipid droplets. The increase in lipid droplets observed in *MIR494* expressing cells was dependent on LC3B. *ATG7* knockdown led to an increase in smaller sized lipid droplets which was not altered by *MIR494* expression

Herein, we now demonstrate that *MIR494* leads to a marked reduction in cell growth in 769-P renal cancer cells which is associated with lipid droplets, reduced total cholesterol levels, and mitochondrial changes. The reduction in cell viability induced by *MIR494* has been observed in other cancer types; indeed, several *MIR494* targets have been thus far identified which are associated with reduced cellular survival including IGF2 [17], c-KIT [20], HOXA10 [32], CLPTM1L [33], SCGN [34], CXCR4 [35], and c-myc [36]. As schematically depicted in Fig. 6, we observed that *MIR494* expression increased lipid droplets in a LC3B–dependent manner. Further, knockdown of ATG7 increased the number of lipid droplets in a *MIR494-*independent manner. Altered mitochondrial patterns and increased LC3B-punctae size were also observed in *MIR494* expressing cells. The effects of *MIR494* were shown to be mediated in an Ago2-dependent mechanism and could also be reversed through the use of antagomirs. However, since Ago2 is an important mediator in miRNA biogenesis pathway, it is likely to affect the expression of multiple other miRNAs. Nonetheless, a similar phenomenon to what we observed has been reported for other targets such as RelA which undergoes a miRNA-activation mechanism that is ablated following Ago2 siRNA treatment [37]. It is interesting that Ago2 is targeted for degradation in the autophagosome via NDP52 and therefore, the autophagic process appears essential in controlling the activity of miRNAs [38].

We also demonstrate that LC3B may be a novel downstream target of *MIR494*. Although we identified that there was a significant and reproducible increase in LC3B 3′-UTR luciferase activity, the physiological importance of this observation remains unclear until additional mutational analyses of the 3′-UTR of LC3B to assess whether LC3B is a direct target of *MIR494* is performed. However, the fold-increase was subtle (although significant and reproducible). In contrast, the LC3B promoter activity was markedly increased (50 %) by *MIR494*. This observation is supported by evidence in the literature describing an RNA activation mechanism involving promoter-based events [37, 39] in which the miRNA has target sites in the promoter regions of the DNA. Since LC3B has been reported to be involved in the formation of lipid droplets [26], it is interesting that *MIR494* expression leads to increased LC3B mRNA and protein. Transcriptional regulation of LC3B is reported to be involved in regulating autophagosome size [40, 41]. As indicated earlier, autophagic proteins such as LC3B and ATG7 have roles in lipid droplet biogenesis [26]. Specifically, the conjugated form of LC3B (LC3-II) is associated with the outer membrane of lipid droplets [26]. The conjugation process of LC3B to phosphatidylethanolamine is contributed by the activity of ATG7, an E1-like activating enzyme; however, LC3B can also associate with membranes independently of ATG7 in some systems [42]. Since we observed that knockdown of ATG7 did not reduce *MIR494* induced formation of lipid droplets, ATG7 may be dispensable for lipid droplet formation under our conditions. In support of our results that small lipid droplets increased with *ATG7* knockdown, cells isolated from mice with a targeted deletion of ATG7 showed increased lipid droplets [27].

Apoptosis is well-known to be accompanied by mitochondrial fragmentation [43]. Indeed, our findings also demonstrate changes in mitochondrial structures. Our observation of reduced PINK1 protein and altered subcellular distribution of Drp1 in *MIR494* expressing cells is similar to a prior report where elevated oxidative stress leads to loss of PINK1 which enhances Drp1 recruitment to mitochondria thus augmenting mitochondrial fission [29, 30]. Interestingly, PINK1 is regulated by PTEN [44] which is a reported target of *MIR494* [45–49]; in this regard, it is possible that down-regulation of PTEN may result in the observed reduction in PINK1 protein.

Since we noted increased localization LC3B to disorganized mitochondria, it may be possible that *MIR494* may lead to changes in the mitophagy pathway although more evidence is required. Indeed, prior findings have implicated PINK1 (as well as Parkin) in modulating selective autophagy of mitochondria [50]. Strikingly, recent evidence indicates that mitochondrial structural deformities can alter the intracellular movement of fatty acids [4, 28]. Under conditions of starvation, fatty acids enter the mitochondrial compartment for β-oxidation; however, decreased mitochondrial fusion events may oppose this process promoting fatty acid movement to the lipid droplets, thereby leading to an accumulation of lipid droplets in the cell [28]. Therefore, our observation of only subtle changes in cellular survival following LC3B siRNA treatment suggests that the formation of lipid droplets may be downstream of the apoptotic/mitochondrial changes. Further experiments need to be conducted to determine whether there is any alteration in mitochondrial β-oxidation or movement of fatty acids from mitochondria to lipid droplets.

Although we identified cholesterol clefts via TEMs in *MIR494* expressing cells which were similar to that reported in atherosclerosis [51], we observed a significant reduction in total cellular cholesterol content upon *MIR494* expression. Since cholesterol disrupting agents such as lovastatin increases cell death [52], a reduction in cholesterol levels induced by *MIR494* expression supports the observation that *MIR494* reduced cellular viability with an increase in the apoptotic response. A few miRNA target genes have thus far been shown to regulate lipid metabolism; these miRNAs include *MIR33*, *MIR122*, *MIR378*, *and MIR125* [53]. Since *MIR494* expression leads to increased lipid droplets, it may be possible that *MIR494* directly target genes involved in the lipid biogenesis pathway.

## Conclusions

Our findings reported herein demonstrate that *MIR494* expression leads to reduced cellular viability in 769-P renal cancer cells. *MIR494* alters cellular survival by increasing apoptosis associated with mitochondrial changes, reduced cholesterol levels, and increased lipid droplet formation in an LC3B-dependent manner.

## Abbreviations

DIO3: type 3 iodothyronine deiodinase (D3)
DLK1: delta-like 1 homolog
Drp1: dynamin-related protein 1
FBS: fetal bovine serum
LC3: microtubule-associated protein 1 light chain 3 alpha
LD: lipid droplets
miRNA: microRNA
NaOH: sodium hydroxide
PARP: poly ADP ribose polymerase
PI: propidium Iodide
PINK1: PTEN-induced putative kinase 1
STR: short tandem repeat
TEM: transmission electron microscopy
UTR: untranslated region

## References

[1] Edinger AL, Thompson CB (2004). Death by design: apoptosis, necrosis and autophagy. Curr Opin Cell Biol.

[2] Zhang L, Huang J, Yang N, Greshock J, Megraw MS, Giannakakis A (2006). microRNAs exhibit high frequency genomic alterations in human cancer. Proc Natl Acad Sci U S A.

[3] Croce CM (2009). Causes and consequences of microRNA dysregulation in cancer. Nat Rev Genet.

[4] Boren J, Brindle KM (2012). Apoptosis-induced mitochondrial dysfunction causes cytoplasmic lipid droplet formation. Cell Death Differ.

[5] Gbelcova H, Sveda M, Laubertova L, Varga I, Vitek L, Kolar M (2013). The effect of simvastatin on lipid droplets accumulation in human embryonic kidney cells and pancreatic cancer cells. Lipids Health Dis.

[6] Goodman JM (2008). The gregarious lipid droplet. J Biol Chem.

[7] DiMarco DM, Fernandez ML (2015). The regulation of reverse cholesterol transport and cellular cholesterol homeostasis by MicroRNAs. Biology (Basel).

[8] Smolle E, Haybaeck J (2014). Non-coding RNAs and lipid metabolism. Int J Mol Sci.

[9] Horie T, Baba O, Kuwabara Y, Yokode M, Kita T, Kimura T (2014). MicroRNAs and lipoprotein metabolism. J Atheroscler Thromb.

[10] Sacco J, Adeli K (2012). MicroRNAs: emerging roles in lipid and lipoprotein metabolism. Curr Opin Lipidol.

[11] Flowers E, Froelicher ES, Aouizerat BE (2013). MicroRNA regulation of lipid metabolism. Metabolism.

[12] Vickers KC, Sethupathy P, Baran-Gale J, Remaley AT (2013). Complexity of microRNA function and the role of isomiRs in lipid homeostasis. J Lipid Res.

[13] Moore KJ, Rayner KJ, Suarez Y, Fernandez-Hernando C (2011). The role of microRNAs in cholesterol efflux and hepatic lipid metabolism. Annu Rev Nutr.

[14] Drabkin HA, Gemmill RM (2010). Obesity, cholesterol, and clear-cell renal cell carcinoma (RCC). Adv Cancer Res.

[15] Benetatos L, Hatzimichael E, Londin E, Vartholomatos G, Loher P, Rigoutsos I (2013). The microRNAs within the DLK1-DIO3 genomic region: involvement in disease pathogenesis. Cell Mol Life Sci.

[16] Haga CL, Phinney DG (2012). MicroRNAs in the imprinted DLK1-DIO3 region repress the epithelial-to-mesenchymal transition by targeting the TWIST1 protein signaling network. J Biol Chem.

[17] Ohdaira H, Sekiguchi M, Miyata K, Yoshida K (2012). MicroRNA-494 suppresses cell proliferation and induces senescence in A549 lung cancer cells. Cell Prolif.

[18] Comegna M, Succoio M, Napolitano M, Vitale M, D’Ambrosio C, Scaloni A (2014). Identification of miR-494 direct targets involved in senescence of human diploid fibroblasts. FASEB J.

[19] Yamanaka S, Campbell NR, An F, Kuo SC, Potter JJ, Mezey E (2012). Coordinated effects of microRNA-494 induce G(2)/M arrest in human cholangiocarcinoma. Cell Cycle.

[20] Kim WK, Park M, Kim YK, Tae YK, Yang HK, Lee JM (2011). MicroRNA-494 downregulates KIT and inhibits gastrointestinal stromal tumor cell proliferation. Clin Cancer Res.

[21] Smith DM, Patel S, Raffoul F, Haller E, Mills GB, Nanjundan M (2010). Arsenic trioxide induces a beclin-1-independent autophagic pathway via modulation of SnoN/SkiL expression in ovarian carcinoma cells. Cell Death Differ.

[22] Liang XH, Hart CE, Crooke ST (1829). Transfection of siRNAs can alter miRNA levels and trigger non-specific protein degradation in mammalian cells. Biochim Biophys Acta.

[23] Liu J, Yang G, Thompson-Lanza JA, Glassman A, Hayes K, Patterson A (2004). A genetically defined model for human ovarian cancer. Cancer Res.

[24] Daugas E, Susin SA, Zamzami N, Ferri KF, Irinopoulou T, Larochette N (2000). Mitochondrio-nuclear translocation of AIF in apoptosis and necrosis. FASEB J.

[25] Liu Y, Chen L, Gong Z, Shen L, Kao C, Hock JM (2015). Lovastatin enhances adenovirus-mediated TRAIL induced apoptosis by depleting cholesterol of lipid rafts and affecting CAR and death receptor expression of prostate cancer cells. Oncotarget.

[26] Shibata M, Yoshimura K, Furuya N, Koike M, Ueno T, Komatsu M (2009). The MAP1-LC3 conjugation system is involved in lipid droplet formation. Biochem Biophys Res Commun.

[27] Guo JY, Karsli-Uzunbas G, Mathew R, Aisner SC, Kamphorst JJ, Strohecker AM (2013). Autophagy suppresses progression of K-ras-induced lung tumors to oncocytomas and maintains lipid homeostasis. Genes Dev.

[28] Rambold AS, Cohen S, Lippincott-Schwartz J (2015). Fatty acid trafficking in starved cells: regulation by lipid droplet lipolysis, autophagy, and mitochondrial fusion dynamics. Dev Cell.

[29] Dagda RK, Cherra SJ, Kulich SM, Tandon A, Park D, Chu CT (2009). Loss of PINK1 function promotes mitophagy through effects on oxidative stress and mitochondrial fission. J Biol Chem.

[30] Lutz AK, Exner N, Fett ME, Schlehe JS, Kloos K, Lammermann K (2009). Loss of parkin or PINK1 function increases Drp1-dependent mitochondrial fragmentation. J Biol Chem.

[31] Chen H, Chan DC (2009). Mitochondrial dynamics--fusion, fission, movement, and mitophagy--in neurodegenerative diseases. Hum Mol Genet.

[32] Liborio-Kimura TN, Jung HM, Chan EK (2015). miR-494 represses HOXA10 expression and inhibits cell proliferation in oral cancer. Oral Oncol.

[33] Zhang R, Chen X, Zhang S, Zhang X, Li T, Liu Z (2015). Upregulation of miR-494 inhibits cell growth and invasion and induces cell apoptosis by targeting cleft Lip and palate transmembrane 1-like in esophageal squamous cell carcinoma. Dig Dis Sci.

[34] Bai Y, Sun Y, Peng J, Liao H, Gao H, Guo Y (2014). Overexpression of secretagogin inhibits cell apoptosis and induces chemoresistance in small cell lung cancer under the regulation of miR-494. Oncotarget.

[35] Shen PF, Chen XQ, Liao YC, Chen N, Zhou Q, Wei Q (2014). MicroRNA-494-3p targets CXCR4 to suppress the proliferation, invasion, and migration of prostate cancer. Prostate.

[36] He W, Li Y, Chen X, Lu L, Tang B, Wang Z (2014). miR-494 acts as an anti-oncogene in gastric carcinoma by targeting c-myc. J Gastroenterol Hepatol.

[37] Dharap A, Pokrzywa C, Murali S, Pandi G, Vemuganti R (2013). MicroRNA miR-324-3p induces promoter-mediated expression of RelA gene. PLoS One.

[38] Gibbings D, Mostowy S, Jay F, Schwab Y, Cossart P, Voinnet O (2012). Selective autophagy degrades DICER and AGO2 and regulates miRNA activity. Nat Cell Biol.

[39] Huang V, Place RF, Portnoy V, Wang J, Qi Z, Jia Z (2012). Upregulation of Cyclin B1 by miRNA and its implications in cancer. Nucleic Acids Res.

[40] Backues SK, Lynch-Day MA, Klionsky DJ (2012). The Ume6-Sin3-Rpd3 complex regulates ATG8 transcription to control autophagosome size. Autophagy.

[41] Jin M, Klionsky DJ (2014). Regulation of autophagy: modulation of the size and number of autophagosomes. FEBS Lett.

[42] Singh R, Cuervo AM (2012). Lipophagy: connecting autophagy and lipid metabolism. Int J Cell Biol.

[43] Suen DF, Norris KL, Youle RJ (2008). Mitochondrial dynamics and apoptosis. Genes Dev.

[44] O’Flanagan CH, O’Neill C (1846). PINK1 signalling in cancer biology. Biochim Biophys Acta.

[45] Liu K, Liu S, Zhang W, Jia B, Tan L, Jin Z (2015). miR-494 promotes cell proliferation, migration and invasion, and increased sorafenib resistance in hepatocellular carcinoma by targeting PTEN. Oncol Rep.

[46] Chen HH, Huang WT, Yang LW, Lin CW (2015). The PTEN-AKT-mTOR/RICTOR pathway in nasal natural killer cell lymphoma is activated by miR-494-3p via PTEN but inhibited by miR-142-3p via RICTOR. Am J Pathol.

[47] Wang J, Chen H, Liao Y, Chen N, Liu T, Zhang H (2015). Expression and clinical evidence of miR-494 and PTEN in non-small cell lung cancer. Tumour Biol.

[48] Li XT, Wang HZ, Wu ZW, Yang TQ, Zhao ZH, Chen GL (2015). miR-494-3p regulates cellular proliferation, invasion, migration, and apoptosis by PTEN/AKT signaling in human glioblastoma cells. Cell Mol Neurobiol.

[49] Liu L, Jiang Y, Zhang H, Greenlee AR, Han Z (2010). Overexpressed miR-494 down-regulates PTEN gene expression in cells transformed by anti-benzo(a)pyrene-trans-7,8-dihydrodiol-9,10-epoxide. Life Sci.

[50] Eiyama A, Okamoto K (2015). PINK1/Parkin-mediated mitophagy in mammalian cells. Curr Opin Cell Biol.

[51] Guyton JR, Klemp KF (1993). Transitional features in human atherosclerosis. Intimal thickening, cholesterol clefts, and cell loss in human aortic fatty streaks. Am J Pathol.

[52] Iimura O, Vrtovsnik F, Terzi F, Friedlander G (1997). HMG-CoA reductase inhibitors induce apoptosis in mouse proximal tubular cells in primary culture. Kidney Int.

[53] Fernandez-Hernando C, Suarez Y, Rayner KJ, Moore KJ (2011). MicroRNAs in lipid metabolism. Curr Opin Lipidol.

